# Pancreatic Ductal Adenocarcinoma at CT: A Combined Nomogram Model to Preoperatively Predict Cancer Stage and Survival Outcome

**DOI:** 10.3389/fonc.2021.594510

**Published:** 2021-05-24

**Authors:** Chunyuan Cen, Liying Liu, Xin Li, Ailan Wu, Huan Liu, Xinrong Wang, Heshui Wu, Chunyou Wang, Ping Han, Siqi Wang

**Affiliations:** ^1^ Department of Radiology, Union Hospital, Tongji Medical College, Huazhong University of Science and Technology, Wuhan, China; ^2^ Hubei Province Key Laboratory of Molecular Imaging, Wuhan, China; ^3^ Department of Radiology, The First Affiliated Hospital, College of Medicine, Zhejiang University, Hangzhou, China; ^4^ Advanced Application Team, GE Healthcare, Shanghai, China; ^5^ Translational Medicine Team, GE Healthcare, Shanghai, China; ^6^ Department of Pancreatic Surgery, Union Hospital, Tongji Medical College, Huazhong University of Science and Technology, Wuhan, China

**Keywords:** pancreatic ductal adenocarcinoma, computed tomography, radiomics, nomogram, cancer staging

## Abstract

**Objectives:**

To construct a nomogram model that combines clinical characteristics and radiomics signatures to preoperatively discriminate pancreatic ductal adenocarcinoma (PDAC) in stage I-II and III-IV and predict overall survival.

**Methods:**

A total of 135 patients with histopathologically confirmed PDAC who underwent contrast-enhanced CT were included. A total of 384 radiomics features were extracted from arterial phase (AP) or portal venous phase (PVP) images. Four steps were used for feature selection, and multivariable logistic regression analysis were used to build radiomics signatures and combined nomogram model. Performance of the proposed model was assessed by using receiver operating characteristic (ROC) curves, calibration curves and decision curve analysis (DCA). Kaplan-Meier analysis was applied to analyze overall survival in the stage I-II and III-IV PDAC groups.

**Results:**

The AP+PVP radiomics signature showed the best performance among the three radiomics signatures [training cohort: area under the curve (AUC) = 0.919; validation cohort: AUC = 0.831]. The combined nomogram model integrating AP+PVP radiomics signature with clinical characteristics (tumor location, carcinoembryonic antigen level, and tumor maximum diameter) demonstrated the best discrimination performance (training cohort: AUC = 0.940; validation cohort: AUC = 0.912). Calibration curves and DCA verified the clinical usefulness of the combined nomogram model. Kaplan-Meier analysis showed that overall survival of patients in the predicted stage I-II PDAC group was longer than patients in stage III-IV PDAC group (p<0.0001).

**Conclusions:**

We propose a combined model with excellent performance for the preoperative, individualized, noninvasive discrimination of stage I-II and III-IV PDAC and prediction of overall survival.

## Introduction

Pancreatic cancer is the fourth most common cause of cancer-related death in the United States, with a 5-year survival rate of 9.3% (1). The number of new pancreatic cancer cases in the United States is expected to reach 56,770, with 45,750 deaths, by the end of 2019 (2). Pancreatic ductal adenocarcinoma (PDAC) is the predominant histological subtype, accounting for 85% of pancreatic malignancies (3). Currently, complete surgical resection is the only potentially curative treatment for PDAC. However, owing to the lack of typical symptoms and physical signs, more than 80% of patients with PDAC are identified in the advanced stages and have missed the opportunity for optimal radical surgery (4). In pancreatic cancer, approximately 10.3% of patients are diagnosed at the local stage and have a 5-year survival rate of 37.4%, while approximately 53% of patients have metastasized when diagnosed, with a 5-year survival rate of only 2.9% (2). Therefore, accurate cancer staging plays a critical role in predicting prognosis and choosing a suitable treatment option for patients with PDAC. However, for most PDAC patients, an accurate cancer stage can be confirmed only by a postoperative histopathologic examination; therefore, a preoperative, noninvasive and accurate method is still urgently needed.

Due to its superior spatial resolution, low costs, and widespread availability, multidetector computed tomography (MDCT) is the first-line imaging modality for the initial evaluation of suspected PDAC (5). Radiomics, which enables the extraction of high-throughput imaging features from medical images, is an emerging field that provides a noninvasive quantitative method for cancer diagnosis, staging, and the evaluation of curative effects (6, 7). Previous studies have demonstrated advancement in the preoperative prediction of cancer stage by applying a radiomics-based approach in esophageal cancer (8), colorectal cancer (9), and head and neck squamous cell carcinoma (10). Eilaghi et al. suggested that CT-derived PDAC texture features were correlated with overall survival and disease-free survival in patients undergoing resection (11). Cassinotto et al. demonstrated that resectable pancreatic adenocarcinoma attenuation parameters on CT scans had a significant association with tumor differentiation grade, lymph node invasion, and disease-free survival (12). Bian et al. (13) concluded that arterial radiomics score is independently and positively associated with the risk of lymph node metastasis in PDAC.

The aim of this study was to construct a combined nomogram model that incorporates radiomics signatures based on contrast-enhanced CT arterial phase (AP) and portal vein phase (PVP) images with clinical factors to preoperatively predict PDAC stage (I-II or III-IV) and survival.

## Materials and Methods

### Patients

**Figure 1:**
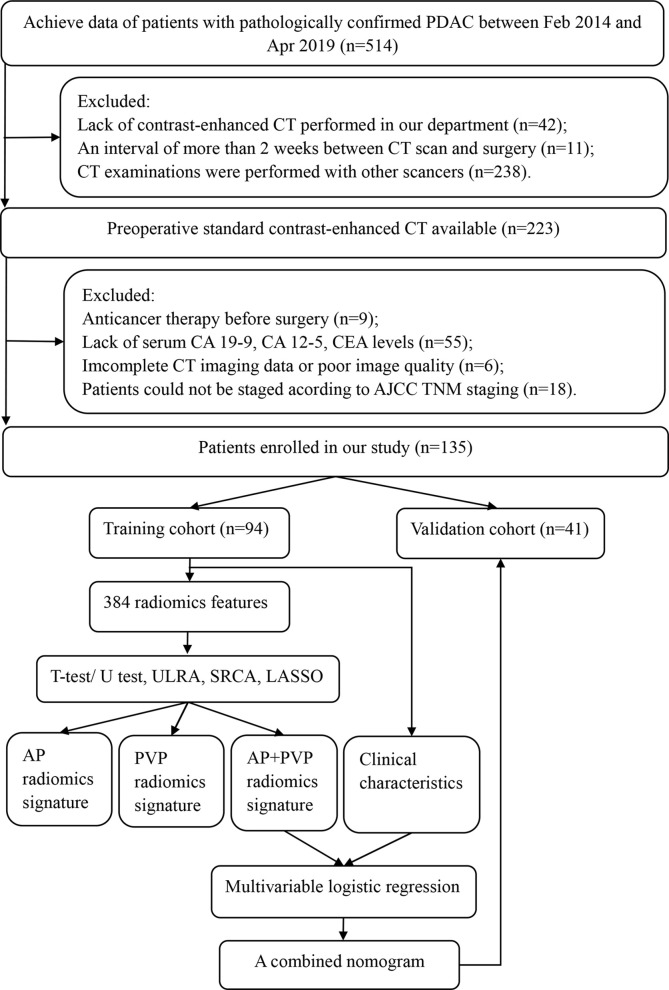
Framework of this study. ULRA, univariate logistic regression analysis; SRCA, Spearman rank correlation analysis; LASSO, least absolute shrinkage and selection operator; AP, arterial phase; PVP, portal vein phase.

This retrospective study was approved by the ethical committee of Tongji Medical College, Huazhong University of Science and Technology, which was in accordance with the Declaration of Helsinki. The requirement for written informed consent was waived. Patient data were collected from the institutional database between February 2014 and April 2019. The inclusion criteria were as follows: (a) histopathological diagnosis of PDAC, including total pancreatectomy, pancreaticoduodenectomy, distal pancreatectomy, laparoscopic biopsy, and exploratory laparotomy biopsy; (b) standard contrast-enhanced CT performed <2 weeks before surgery, and (c) CT examination was performed with a SOMATOM Definition AS+ scanner (Siemens Healthcare, Erlangen, Germany). The exclusion criteria were as follows: (a) any other anticancer therapy prior to surgery, such as chemotherapy, radiotherapy, or immunotherapy; (b) incomplete clinical information; (c) incomplete CT imaging data or poor image quality; or (d) could not be staged according to American Joint Committee on Cancer (AJCC) TNM staging. The patient selection workflow and model construction framework are shown in **Figure 1**. Two abdominal radiologists with 10 years and 6 years of experience reviewed all CT images and extracted the maximum tumor diameter and tumor location in all patients. Clinical data [age, gender, preoperative carbohydrate antigen 19-9 (CA 19-9), carbohydrate antigen 12-5 (CA12-5), and carcinoembryonic antigen (CEA) level], the status of vascular involvement observed during surgery, the status of pathologically confirmed lymph node metastasis, and histopathological data were acquired from medical records. Tumor staging was determined according to the AJCC TNM Staging System Manual, 8th Edition (14). Overall survival data of patients with PDAC were acquired through clinical follow-up and telephone communications.

### CT Image Acquisition

CT examination was performed on a SOMATOM Definition AS+ scanner (Siemens Healthcare, Erlangen, Germany) with the following parameters: 120 kVp; variable tube current (160-600 mA) depending on the size of the patient; detector collimation, 128×0.6 mm; algorithm, B30; reconstructed thicknesses, 2.0 mm; and increments, 2 mm. After unenhanced scanning, approximately 65-75 mL of iohexol (350 mg I/mL, Omnipaque, GE Healthcare) was injected into the antecubital vein at 2.0-2.5 mL/s *via* a pump injector. CT scans of the AP and PVP were carried out at 25-35 s and 60-70 s after injection, respectively.

### Region-of-Interest (ROI) Segmentation, Radiomics Feature Extraction, and Intra-and Interobserver Reproducibility

**Figure 2:**
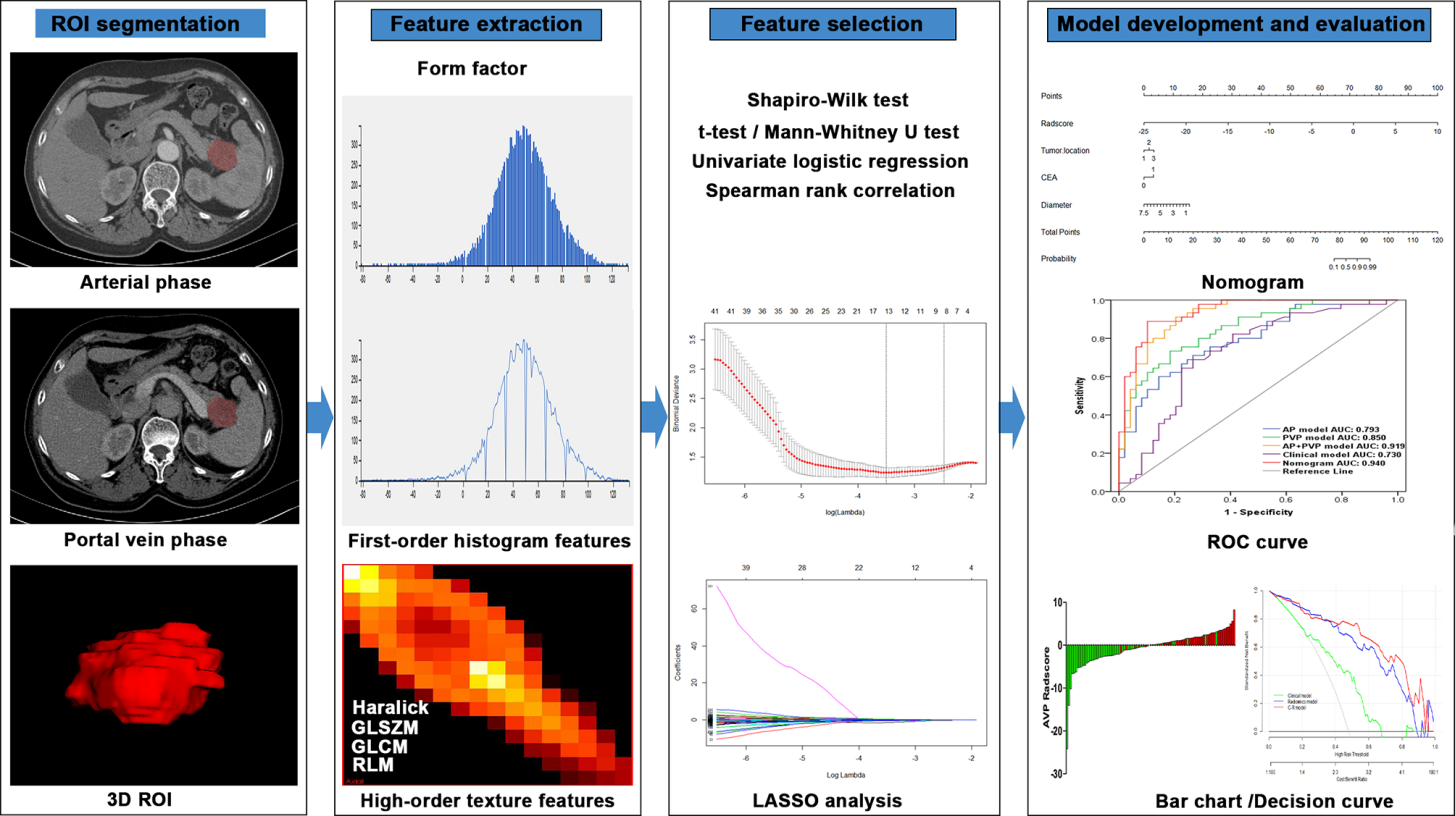
Flowchart of the radiomics method for PDAC stage prediction. LASSO, least absolute shrinkage and selection operator; ROC, receiver operating characteristic.

The workflow of radiomics analysis was shown in **Figure 2**. The 3D ROI of the tumor was manually contoured on AP and PVP CT images using ITK-SNAP software (15). The ROIs of all patients were contoured by two radiologists (X.L., with 10 years of expertise in abdominal imaging diagnosis, and S.W., with 6 years of expertise in abdominal imaging diagnosis); both were blinded to the pathological results.

To assess potential differences in tumor segmentation between radiologists, the intra- and interclass correlation coefficients (ICCs) were used to evaluate the differences between features generated by SW (first time) and those generated by X.L. and between features generated twice by SW (16). ICCs were classified as follows: 0-0.2, no agreement; 0.21-0.40, weak agreement; 0.41-0.60, moderate agreement; 0.61-0.80, good agreement; and 0.81-1, excellent agreement.

Using in-house software (Analysis Kit, version 3.1.5.R, GE Healthcare, China), 384 radiomics features were extracted from 3D ROIs. The extracted radiomics features included 42 histogram features, 132 gray-level cooccurrence matrix features, 11 gray-level size zone matrix features, 180 gray-level run-length matrix features, 10 Haralick features, and 9 form factor features.

### Radiomics Feature Selection and Radiomics Signature Construction

First, the Shapiro-Wilk test was used to examine the normality of feature distribution. A t-test/Mann-Whitney U test was used to analyze significant differences between stage I-II and III-IV depending on feature distribution. Second, a univariate logistic regression analysis was performed to investigate associations of single features with cancer stage. Third, a Spearman rank correlation analysis was performed to remove correlated features with correlation coefficients greater than 0.9. Finally, least absolute shrinkage and selection operator (LASSO) analysis was applied for dimension reduction and selection of the most informative features from the remainder of the features. LASSO analysis with penalty tuning parameters (lambda value) was used to select significant features for the model, which was conducted by applying 10-fold cross-validation based on the minimum criteria. Nonzero coefficient variables were selected by LASSO, while most covariate coefficients decreased to zero. Radiomics scores (rad-scores) were calculated for each patient based on the AP, PVP, and AP+PVP radiomics signatures to better evaluate the performance of the signature. The signatures were constructed using coefficients weighted by the LASSO logistic regression model in the training cohort. We also assessed the differences in rad-scores between stage I-II and III-IV in the training and validation cohorts.

### Development of the Clinical Model and the Combined Nomogram Model

Clinical and combined models were also built for comparison with the radiomics model. Univariate and multivariate analyses were applied to find out independent clinical characteristics, which were used to develop the clinical model for predicting cancer stage. The proposed radiomics signature and the independent clinical characteristics were integrated by multivariable logistic regression analysis to construct the combined nomogram, which can provide a quantitative tool to differentiate stage I-II and III-IV PDAC.

### Performance and Validation of the Combined Nomogram Model

The model performances were evaluated in the validation cohort from three aspects: discrimination, calibration and clinical utility. The discrimination ability of each proposed model was evaluated by a receiver operating characteristic (ROC) curve, area under the curve (AUC), sensitivity, and specificity (17). A calibration curve was drawn *via* bootstrapping with 1000 resamples to evaluate the calibration of the proposed model and assessed by the Hosmer-Lemeshow test. A significant statistic from the test indicated that the model had a poor fit. The predictive accuracy of the proposed model was reflected by the overlap between the calibration curve and the diagonal in the figure. The Decision curve analysis (DCA) was used to quantify the net benefits from the use of the clinical model, radiomics model, and combined nomogram model at different threshold probabilities in the validation cohort (18).

### Survival Analysis

Overall survival was calculated from the date of surgery to the date of death as a result of PDAC or censored at the date of December 24, 2019, or the date of the last observation for surviving patients. Survival analysis was performed to explore the potential of the combined nomogram model to predict overall survival. Patients from the training and validation cohorts were divided into predicted stage I-II and III-IV according to the threshold calculated from the Youden index in training cohort. The Kaplan-Meier curves and log-rank tests were used to analyze the survival of patients with predicted stage I-II and III-IV.

### Statistical Analysis

Categorical variables, such as sex, tumor location, CA19-9 level, CA12-5 level, and CEA level, were analyzed by chi-square test or Fisher’s exact test. Continuous variables, including age, maximum tumor diameter, and rad-score, were analyzed by Student’s t-test or the Mann-Whitney U test, when appropriate. Variables that reached statistical significance in the univariate analysis were included in the combined nomogram. AUC difference between training and validation cohorts was analyzed using the DeLong test. All statistical tests used in this study were executed with R software V 3.6.1 (R Core Team, Vienna, Austria) or SPSS 19.0 statistical software (SPSS, Inc., Chicago, IL, USA). P value < 0.05 was considered statistically significant.

## Results

### Patient Characteristics

A total of 135 patients (87 men and 48 women; mean age, 59.96 ± 9.25 years, age range, 33–78 years) were enrolled in the current study. The characteristics of all patients are shown in **Table 1**. Based on pathological results, PDAC stage was determined according to the AJCC TNM Staging System Manual, 8th Edition. There were 12 patients in stage I A, 18 patients in stage I B, 9 patients in stage II A, 30 patients in stage II B, 19 patients in stage III, and 47 patients in stage IV. Patients were randomly allocated to the training (n = 94) or validation (n = 41) cohort at a ratio of 7:3. No significant difference in clinical characteristics (age, gender, tumor location, and preoperative CA19-9 level, CA 12-5 level, CEA level, tumor maximum diameter) was found between the training and validation cohorts (**Supplementary Table S1**). However, a few clinical characteristics, including tumor location, CEA level, and tumor maximum diameter, were significantly different between patients with stage I-II and III-IV PDAC in the training cohort (**Table 1**); all of these clinical characteristics were included in the clinical predictive model.

**Table 1:** Characteristics of patients in the training and validation cohorts.

Characteristic	Training Cohort (n = 94)		*P* Value	Validation Cohort (n = 41)		*P Value*
	Early stage (n = 49)	Advanced stage (n = 45)		Early stage(n = 21)	Advanced stage (n = 20)	
Age (years), median (range)	60 (33-77)	60 (39-72)	0.928	58 (39-78)	58 (47-65)	0.488
Gender (%)			0.111			0.541
Male	27 (62.8%)	32		14 (68.3%)	14	
Female	22 (37.2%)	13		7 (31.7%)	6	
Tumor location (%)			0.0037*			0.816
Head of pancreas	38 (60.6%)	19		15 (61.0%)	10	
Body of pancreas	4 (19.1%)	14		4 (21.9%)	5	
Tail of pancreas	7 (20.2%)	12		2 (17.1%)	5	
Maximum diameter (cm), median (range)	2.8 (0.8-6.9)	3.9 (1.2-7.4)	0.0024*	2.8 (1.4-10)	3.45 (1.9-10.1)	0.318
CA19-9 (%)			0.363			0.977
<37	15 (26.6%)	10		6 (26.8%)	5	
≥37	34 (73.4%)	35		15 (71.2%)	15	
CA12-5 (%)			0.0615			0.496
<35	28 (47.9%)	17		9 (41.5%)	8	
≥35	21 (52.1%)	28		12 (58.5%)	12	
CEA level (%)			0.0434*			0.731
<5	35 (61.7%)	23		11 (58.5%)	13	
≥5	14 (38.3%)	22		10 (41.5%)	7	
AP Rad-score (mean ± SD)	-0.8174 ± 0.1922	0.6514 ± 0.2005	<0.0001*	-0.2655 ± 0.2666	0.8453 ± 0.4531	0.0389*
PVP Rad-score (mean ± SD)	-1.333 ± 0.2786	1.719 ± 0.6186	<0.0001*	-0.8151 ± 0.6176	0.7543 ± 0.3631	0.0367*
AP+PVP Rad-score (mean ± SD)	-0.8174 ± 0.1922	0.6514 ± 0.2005	<0.0001*	-2.813 ± 0.6681	0.2171 ± 1.162	0.0277*
Clinic model score (mean ± SD)	0.4113 ± 0.02563	0.5521 ± 0.02489	0.0002*	0.4238 ± 0.03181	0.5550 ± 0.04277	0.0176*
Nomogram Rad-score (mean ± SD)	0.1854 ± 0.03625	0.7982 ± 0.03571	<0.0001*	0.2258 ± 0.05102	0.7629 ± 0.06029	<0.0001*

CA19-9, carbohydrate antigen 19-9; CA12-5, carbohydrate antigen 12-5; CEA, carcinoembryonic antigen; AP, arterial phase; PVP, portal vein phase; Rad-score, radiomics score. *P < 0.05.

### Radiomics Feature Selection and Radiomics Signature Construction

**Figure 3:**
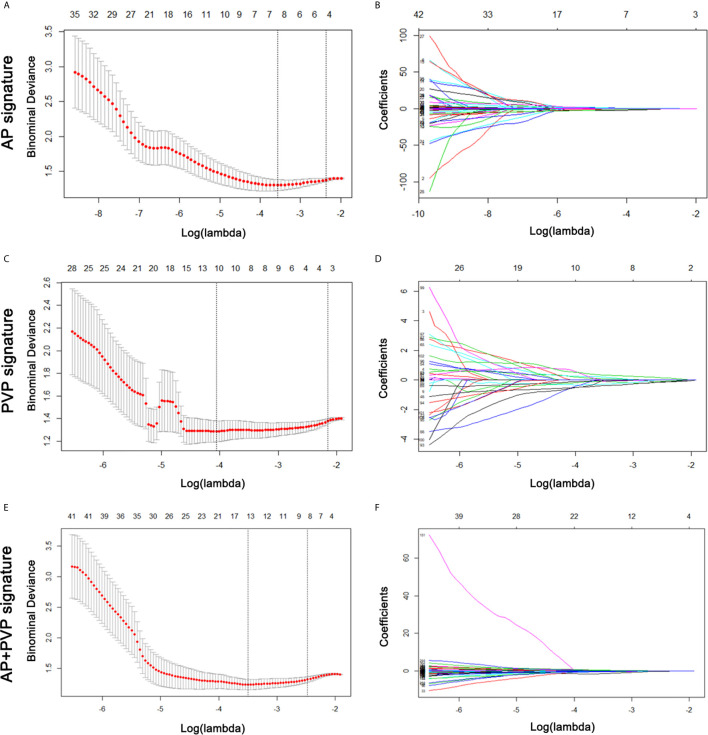
AP, PVP, and AP+PVP radiomics feature selection by LASSO regression. **(A, C, E)** Selection of tuning parameters (lambda value) in the LASSO model using ten-fold cross-validation by the minimum criteria. **(B, D, F)** LASSO coefficient profiles of the radiomics features. LASSO, least absolute shrinkage and selection operator; AP, arterial phase; PVP, portal vein phase.

From the training cohort, 384 radiomics features were extracted based on AP and PVP CT images. For the AP+PVP signature construction, 384 AP radiomics features and 384 PVP radiomics features were included. The mean interobserver correlation coefficients were 0.858 and 0.944 for the 384 AP and 384 PVP radiomics features, respectively. The mean intraclass correlation coefficients were 0.761 and 0.901 for the 384 AP and 384 PVP radiomics features, respectively. The lambda value with the minimum criteria in the LASSO model using 10-fold cross-validation was chosen (**Figure 3**). Finally, 8 AP, 10 PVP and 14 AP+PVP radiomics features were confirmed for AP, PVP, and AP+PVP radiomics signatures, and formulas for the rad-scores were generated through a linear combination of these features weighted by the LASSO algorithm. Each feature’s coefficient was calculated from the LASSO regression method (**Supplementary Table S2**). Details of the rad-score formulas are shown in **Supplementary I**.

### Diagnostic Validation of the Radiomics Signature

**Figure 4:**
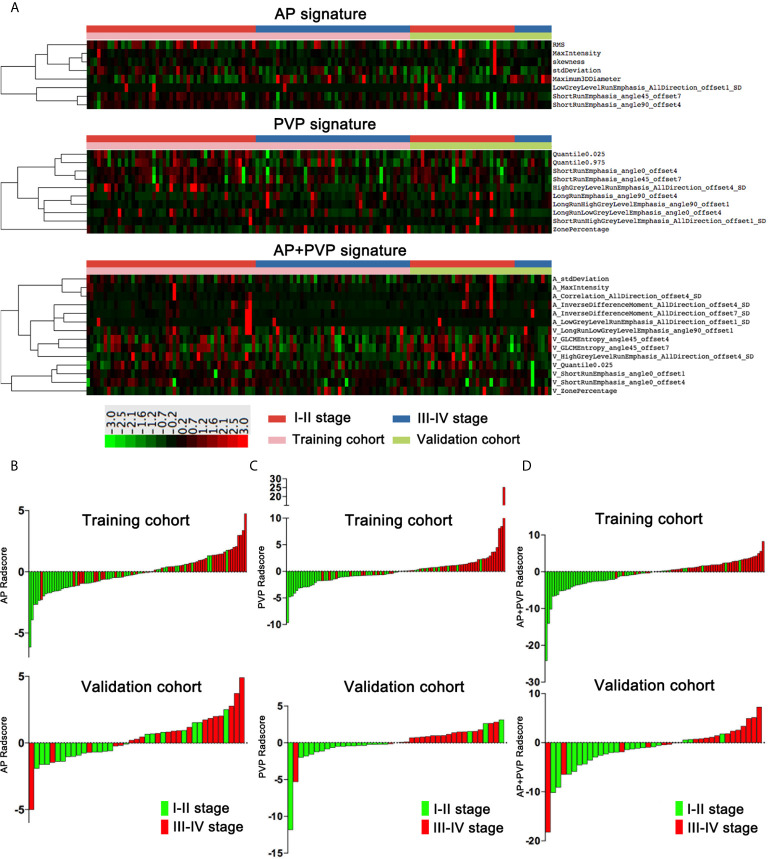
Rad-scores of the AP, PVP, and AP+PVP signatures. **(A)** Heatmap of 8, 10, and 14 radiomics features in the AP, PVP, and AP+PVP signatures, respectively. Each row corresponds to one radiomics feature, and each column corresponds to one patient. The heatmap is grouped according to the stage I-II versus III-IV stage groups in training and validation cohorts. The leftmost lines represent hierarchical clustering of radiomics features, shown as a dendrogram. **(B–D)** AP, PVP, and AP+PVP rad-score of each patient in the training and validation cohorts. rad-score, radiomics score; AP, arterial phase; PVP, portal vein phase.

**Figure 5:**
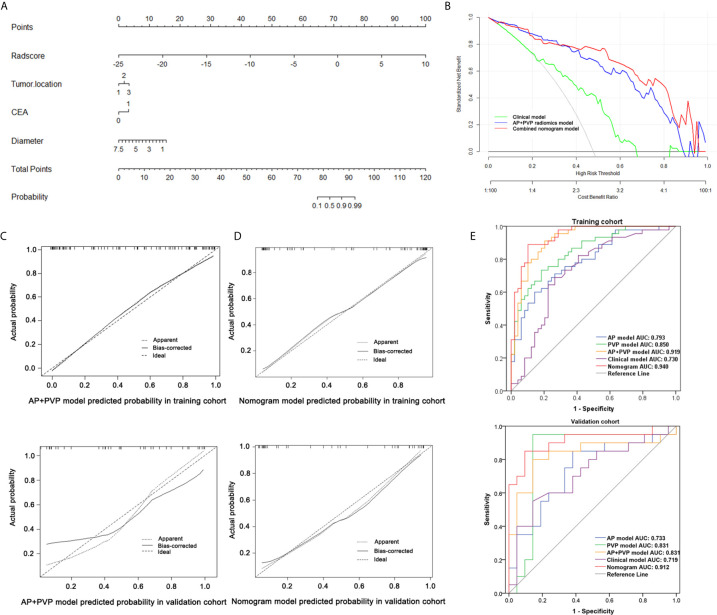
Performance of the combined nomogram and radiomics models. **(A)** Combined nomogram based on three clinical predictors and the AP+PVP radiomics signature. **(B)** Decision curve of the combined nomogram. **(C)** Calibration curve of the AP+PVP radiomics model in the training and validation cohorts. **(D)** Calibration curve of the combined nomogram in the training and validation cohorts. **(E)** ROC curves of the AP, PVP, AP+PVP, clinical, and combined nomogram models in the training and validation cohorts. AP, arterial phase; PVP, portal vein phase; ROC, receiver operating characteristic.

There was a significant difference in the AP, PVP and AP+PVP rad-scores between stage I-II and III-IV PDAC patients in the training and validation cohorts (**Table 1**). The heatmap is grouped according to the stage I-II versus III-IV stage groups in training and validation cohorts (**Figure 4A**). The distributions of rad-scores and cancer stage of each patient in training and validation cohorts are shown in **Figures 4B–D**. ROC curves showed that the AP+PVP radiomics signature performed better in differentiating stage I-II and III-IV PDAC in the training [AUC = 0.919: 95% confidence interval (CI), 0.865 to 0.974] and validation (AUC = 0.831: 95% CI, 0.69 to 0.972) cohorts than the AP radiomics signature (training cohort: AUC = 0.793, 95% CI, 0.697 to 0.869; validation cohort: AUC = 0.733, 95% CI, 0.5772 to 0.859) and the PVP radiomics signature (training cohort: AUC = 0.850, 95% CI, 0.774 to 0.925; validation cohort: AUC = 0.831, 95% CI, 0.676 to 0.986). ROC curves are shown in **Figure 5E**. AUC, sensitivity, and specificity of models are shown in **Table 2**.

**Table 2:** Performance of the radiomics signatures, clinical model, and combined nomogram model.

Methods	Training cohort (n = 94)			Validation cohort (n = 41)			DeLong test
	AUC (95%CI)	SEN	SPE	AUC (95%CI)	SEN	SPE	p-value
AP signature	0.793 (0.697-0.869)	0.600	0.857	0.733 (0.572-0.859)	0.850	0.619	0.527
PVP signature	0.850 (0.774-0.925)	0.733	0.816	0.831 (0.676-0.986)	0.950	0.857	0.830
AP+PVP signature	0.919 (0.865-0.974)	0.911	0.796	0.831 (0.69-0.972)	0.800	0.857	0.257
Clinical model	0.730 (0.629-0.817)	0.689	0.735	0.719 (0.557-0.848)	0.550	0.857	0.910
Combined nomogram	0.940 (0.871-0.979)	0.889	0.898	0.912 (0.781-0.978)	0.850	0.905	0.605

AP, arterial phase; PVP, portal vein phase; AUC, area under the receiver operating characteristic curve; SEN, sensitivity; SPE, specificity.

### Development, Performance, and Validation of the Combined Nomogram

According to the univariate analysis in the training cohort, tumor location, CEA level and tumor maximum diameter were independent clinical characteristics (**Supplementary Table S3**). We entered these clinical characteristics into the multivariable logistic regression analysis to construct a clinical prediction model of cancer stage.

Considering the AP+PVP radiomics signature had the best ability to discriminate stage I-II and III-IV PDAC, the combined nomogram incorporated the AP+PVP radiomics signature and the clinical prediction model (**Figure 5A**). In the training cohort, the combined nomogram yielded the highest discrimination between stage I-II and III-IV PDAC, with an AUC of 0.940 (95% CI: 0.871 to 0.979); the observed AUC value was higher than that of the AP+PVP radiomics signature alone (AUC = 0.919: 95% CI, 0.865 to 0.974) and the clinical prediction model alone (AUC = 0.730: 95% CI, 0.629 to 0.817). In the validation cohort, both the combined nomogram (AUC = 0.912; 95% CI, 0.781 to 0.978) and AP+PVP radiomics signature alone (AUC = 0.831: 95% CI, 0.690 to 0.848) also showed a higher AUC than the clinical prediction model (AUC = 0.719: 95% CI, 0.557 to 0.817).

The calibration curve of both the AP+PVP radiomics signature and the combined nomogram demonstrated good agreement between the nomogram prediction and actual observations of stage I-II and III-IV PDAC (**Figures 5C, D**). For the AP+PVP radiomics signature, the Hosmer-Lemeshow test yielded P values of 0.69 and 0.092 in the training and validation cohorts, respectively, indicating no departure from good fit. For the combined nomogram, the Hosmer-Lemeshow test yielded P values of 0.426 and 0.505 in the training and validation cohorts, respectively, suggesting a perfect fit of the nomogram.

The results of the DCA derived from clinical prediction model, AP+PVP radiomics model, and combined nomogram are shown in **Figure 5B**. The AP+PVP radiomics model and combined nomogram provided better net benefit to predict cancer stage than the clinical model with almost all of the threshold probabilities.

### Survival Analysis

**Figure 6:**
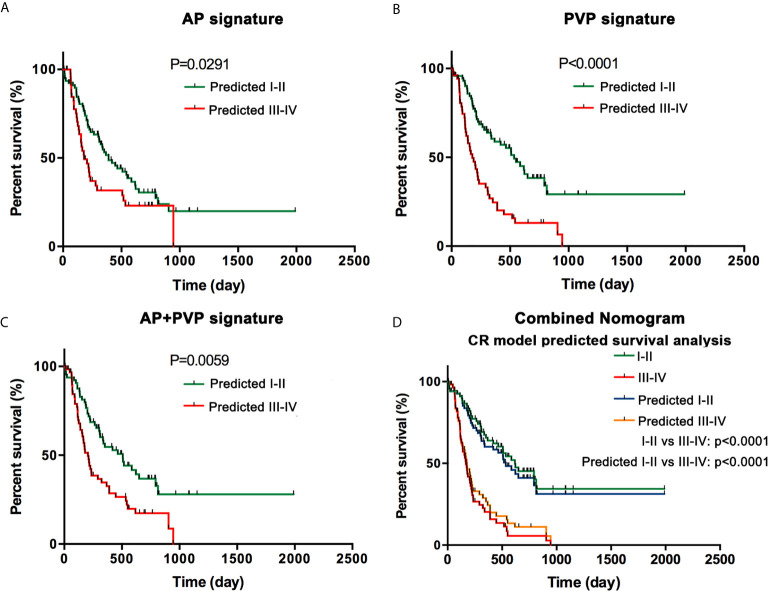
Survival analysis. **(A)** Kaplan-Meier curves in the AP model. **(B)** Kaplan-Meier curves in the PVP model. **(C)** Kaplan-Meier curves in the AP+PVP model. **(D)** Kaplan-Meier curves using histopathological cancer stage and nomogram model-predicted cancer stage. Survival analyses show significant differences between the predicted stage I-II and III-IV groups. AP, arterial phase; PVP, portal vein phase.

Through clinical follow-up and telephone communications, 127 patients were successfully followed up. A total of 84 patients (66.14%) were confirmed deceased, and their survival time ranged from 11 days to 218 days. In the AP, PVP, and AP+PVP radiomics models and the combined nomogram model, Kaplan-Meier survival analysis indicated a significant difference between the predicted stage I-II and III-IV PDAC, suggesting the prognostic value of these models (p = 0.0291, p < 0.0001, p = 0.0059, and p < 0.0001, respectively). Kaplan-Meier curves are shown in **Figure 6**.

## Discussion

In this study, we constructed a combined nomogram that integrates the AP+PVP radiomics signature and clinical characteristics, including tumor location, tumor maximum diameter, and CEA level. In addition, cancer stage predicted by the radiomics model can be a predictor of overall survival, thereby providing important information for clinical decision-making.

Complete surgical resection is the only potentially curative treatment option for PDAC. Unfortunately, only a small number of patients with early-stage PDAC can undergo curative resection. Accurate PDAC staging plays a crucial role in determining resectability and predicting prognosis (19). However, for most PDAC patients, an accurate stage can be obtained only through a histopathological examination after surgery. For clearly localized early-stage PDAC, clinical stage can be determined by MDCT, while for borderline resectable tumors, the determination of clinical stage often requires postoperative pathology (e.g., the N-category, which is stratified according to surgical resection and assessment by histopathology). Endoscopic ultrasound-guided fine needle aspiration (EUS-FNA) is one of the standard procedures for pancreatic cancer diagnosis (20). Hewitt et al. (21) performed a meta-analysis of 4984 patients and demonstrated a pooled sensitivity of 0.85 and a specificity of 0.98 for malignant cytology. Reports have shown that the accuracies of T-staging by EUS range from 62-94%, and those of N-staging range from 50-86% (22). Although EUS-FNA provides a valuable means for pancreatic cancer diagnosis, it shows a poor staging performance, and it is invasive and limited to the detection location of the tumor. The proposed combined nomogram for PDAC staging is noninvasive, easy to use, and highly accurate. Previous research has shown that CA 19-9 serum levels have a sensitivity of 79-81% and a specificity of 82-90% for the diagnosis of PDAC in symptomatic patients (23). Several studies have used CA 19-9 serum levels to predict pancreatic cancer stage and found that CA 19-9 serum levels are significantly different in stage I–IV (24, 25). However, limitations exist, including nonspecific expression, false positive results in the presence of obstructive jaundice, and false negative results in the Lewis-negative genotype (26).

CEA level is sensitive to stage I and II diseases and is associated with tumor metastasis and the treatment response (27). In addition, compared to PDAC in the pancreatic head, PDAC in the pancreatic body or tail is larger, more prone to metastasis and less resectable (28). The preoperative CT-based maximum tumor diameter can be easily obtained. Therefore, we integrated tumor location, maximum tumor diameter, and CEA level as candidate factors during the development of the clinical prediction model. After integrating these factors, the AUC of this model was higher than that of the AP+PVP signature or clinical characteristics alone.

Since MDCT has good spatial and temporal resolution with wide anatomic coverage, it is regarded by many medical institutions as the most important preoperative examination for patients with suspicious pancreatic cancer, which is used for comprehensive local and distant disease assessment (29). In a systemic review involving 30 studies with 1554 patients (30), the pooled sensitivity of CT to diagnosis PDAC was 63% (95% CI 58–67%) and the specificity of 92% (95% CI 90–94%). However, MDCT may not detect small pancreatic masses (<1.5 cm) (31), or a primary pancreatic tumor showing isoattenuation (32). This finding has led to the accuracy of classical MDCT being considerably limited for predicting early-stage PDAC. In addition, traditional radiologic diagnosis is a subjective and qualitative preoperative diagnosis made by visual analysis.

Radiomics is a robust, repeatable and noninvasive method to meet the requirements of clinical implementation and is quantitative and objective for measurements of heterogeneity inside the tumor. Previous studies have shown that radiomics can predict histologic grade of pancreatic neuroendocrine tumors (33) and predict pathology in intraductal papillary mucinous neoplasms by integrating clinical factors, radiomics features, and blood-based miRNA expression data (34). In the PDAC field, previous studies have shown that radiomics features were correlated with tumor differentiation grade, lymph node invasion, overall survival, and disease-free survival for patients with PDAC (11–13). In our study, we developed radiomics models based on both AP and PVP images, in contrast to previous studies. We integrated clinical characteristics with the radiomics signature to construct a combined nomogram model. The proposed nomogram showed good discrimination in both the training cohort (AUC = 0.940) and the validation cohort (AUC = 0.912). We also performed survival analysis with Kaplan-Meier curves and log-rank tests, and the results showed that in the AP, PVP, and AP+PVP signatures and the combined nomogram, overall survival was significantly different in PDAC patients with predicted stage I-II and III-IV. The lower the rad-score was, the longer the patients lived.

PDAC is a tumor with low blood supply. In the arterial and portal venous phases, the degree of enhancement of tumor tissue is much lower than that of normal pancreatic tissue. The tumor-to-pancreas contrast difference was greater in the portal venous phase than in the arterial phase. This was the result of greater enhancement of normal pancreas and lower tumor enhancement during the portal venous phase. In different scanning phase, the images that can be observed by the naked eye is different, and the inherent spatial heterogeneity is different. Therefore, the features used to construct models are different. A comparison of the AP and PVP models revealed that the AP model had the lowest AUC (training cohort, AUC: 0.793; validation cohort, AUC: 0.733), and the PVP model (training cohort, AUC: 0.850; validation cohort, AUC: 0.831) had a better diagnostic performance than the AP model. In our study, PVP was scanned at 60-70 s after injecting iohexol, and there was a best visual contrast difference between enhanced pancreatic parenchyma and tumor, which is indicative of hypoattenuation. There is also another advantage during this phase: the peripancreatic arteries are usually well opacified for concomitant evaluation. Fusion of the AP and PVP models provided the best predictive ability among all the radiomics models (35); the AUCs in the AP+PVP model were 0.919 and 0.831 in the training and validation cohorts, respectively. The combined nomogram, which integrated radiomics signature and clinical characteristics, had higher predictive ability (training cohort, AUC: 0.940; validation cohort, AUC: 0.912) than the AP+PVP model. A previous study showed that after the addition of clinical factors, the combined nomogram showed a significant improvement over the radiomics signature alone (33), which is consistent with our results.

Our study has several limitations. First, it was a retrospective study in nature. Second, the proposed models were established based on data obtained from a single center. In addition, genomic data were not included. To address these limitations, we will further prospectively conduct multiscanner and multicenter study and combine the radiomics and clinical models with pathologic and genetic features.

In conclusion, a combined nomogram with favorable accuracy was developed and validated in this study for the noninvasive, preoperative and convenient prediction of cancer stage and prognosis. We believe that the clinical use of this nomogram can not only maximize the survival benefit of patients with stage I-II PDAC but also minimize the morbidity from unnecessary laparotomy or major surgery for patients with stage III-IV. Therefore, our combined nomogram model may assist in clinical decision-making and achieve a good survival outcome.

## Data Availability Statement

The raw data supporting the conclusions of this article will be made available by the authors, without undue reservation.

## Ethics Statement

The studies involving human participants were reviewed and approved by Ethical committee of Tongji Medical College, Huazhong University of Science and Technology. The ethics committee waived the requirement of written informed consent for participation.

## Author Contributions

SW, CC, LL, and PH conceived of and designed the study. CC, LL, XL, AW, CW, HW, and SW collected and assembled all data. CC, LL, HL, XW, and SW performed data analysis. CC, LL, and SW wrote the manuscript. SW and PH revised the manuscript. All authors contributed to the article and approved the submitted version.

## Funding

This study was financially supported by the National Natural Science Foundation of China (81873895 to PH).

## Conflict of Interest

Author HL and XW were employed by company GE Healthcare.

The remaining authors declare that the research was conducted in the absence of any commercial or financial relationships that could be construed as a potential conflict of interest.

## Abbreviations

PDAC, pancreatic ductal adenocarcinoma; CT, computed tomography; AP, arterial phase; PVP, portal venous phase; LASSO, least absolute shrinkage and selection operator; ROC, receiver operating characteristic; DCA, decision curve analysis; AUC, area under the curve; AJCC, American Joint Committee on Cancer; ROI, Region-of-Interest; ICCs, intra- and interclass correlation coefficients; rad-scores, Radiomics scores; CI, confidence interval; EUS-FNA, Endoscopic ultrasound-guided fine needle aspiration; CA19-9, carbohydrate antigen 19-9; CA12-5, carbohydrate antigen 12-5; CEA, carcinoembryonic antigen.
